# Concomitant left atrial appendage closure during left ventricular assist device surgery can reduce ischaemic cerebrovascular accidents

**DOI:** 10.1093/icvts/ivad112

**Published:** 2023-07-08

**Authors:** Hidefumi Nishida, Valluvan Jeevanandam, Christopher Salerno, Atsushi Nemoto, Tae Song, David Onsager, Ann Nguyen, Jonathan Grinstein, Bow Chung, Nitasha Sarswat, Gene Kim, Sean Pinney, Takeyoshi Ota

**Affiliations:** Department of Surgery, University of Chicago Medicine, Chicago, IL, USA; Department of Surgery, University of Chicago Medicine, Chicago, IL, USA; Department of Surgery, University of Chicago Medicine, Chicago, IL, USA; Department of Surgery, University of Chicago Medicine, Chicago, IL, USA; Department of Surgery, University of Chicago Medicine, Chicago, IL, USA; Department of Surgery, University of Chicago Medicine, Chicago, IL, USA; Department of Medicine, University of Chicago Medicine, Chicago, IL, USA; Department of Medicine, University of Chicago Medicine, Chicago, IL, USA; Department of Medicine, University of Chicago Medicine, Chicago, IL, USA; Department of Medicine, University of Chicago Medicine, Chicago, IL, USA; Department of Medicine, University of Chicago Medicine, Chicago, IL, USA; Department of Medicine, University of Chicago Medicine, Chicago, IL, USA; Department of Surgery, University of Chicago Medicine, Chicago, IL, USA

**Keywords:** Left atrial appendage closure, Ischaemic cerebrovascular accident, Left ventricular assist device

## Abstract

**OBJECTIVES:**

It remains unknown if the left atrial appendage closure (LAAC) at the time of left ventricular assist device (LVAD) surgery can reduce ischaemic cerebrovascular accidents.

**METHODS:**

Consecutive 310 patients who underwent LVAD surgery with HeartMate II or 3 between January 2012 and November 2021 were included in this study. The cohort was divided into 2 groups: patients with LAAC (group A) and without LAAC (group B). We compared the clinical outcomes including the incidence of cerebrovascular accident between 2 groups.

**RESULTS:**

Ninety-eight patients were included in group A, and 212 patients in group B. There were no significant differences between 2 groups in age, preoperative CHADS2 score and history of atrial fibrillation. In-hospital mortality did not differ significantly between the 2 groups (group A: 7.1%, group B: 12.3%, *P* = 0.16). Thirty-seven patients (11.9%) experienced ischaemic cerebrovascular accident (5 patients in group A and 32 patients in group B). The cumulative incidence from ischaemic cerebrovascular accidents in group A (5.3% at 12 months and 5.3% at 36 months) was significantly lower than that in group B (8.2% at 12 months and 16.8% at 36 months; *P* = 0.017). In a multivariable competing risk analysis, LAAC was associated with reducing ischaemic cerebrovascular accidents (hazard ratio 0.38, 95% confidence interval 0.15–0.97, *P* = 0.043).

**CONCLUSIONS:**

Concomitant LAAC in LVAD surgery can reduce ischaemic cerebrovascular accidents without increasing perioperative mortality and complications.

**Figure:**
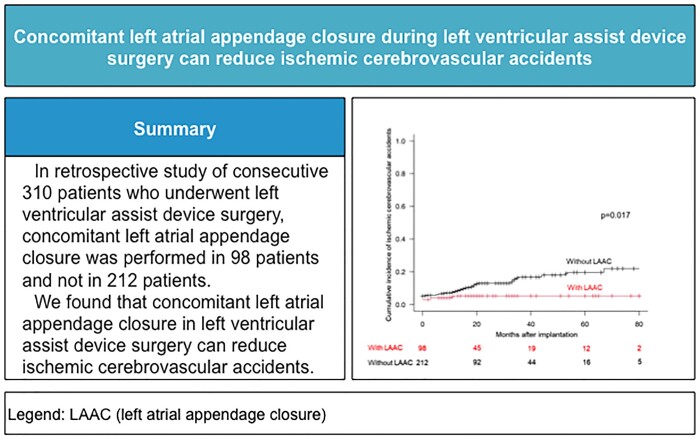

## INTRODUCTION

Left ventricular assist device (LVAD) has emerged as an important treatment strategy for advanced heart failure patients including those who are not candidates for heart transplantation. However, despite advances of technology, cerebrovascular accident remains one of the most significant complications in LVAD patients. The incidence of cerebrovascular accidents in LVAD patients has been reported in the range from 8.0% to 20% [1–3]. A number of clinical studies demonstrated the risk factors for cerebrovascular accident included age, gender, a history of atrial fibrillation, implanted LVAD type, pump thrombosis and intracardiac thrombus [2–7]. Causes of cerebrovascular accidents could be multifactorial and its prevention would not be so simple.

The left atrial appendage (LAA) is well known as a source of thrombus that causes an ischaemic cerebrovascular accident in patients with atrial fibrillation. It is also important to note that LAA could also be a source of thrombus in heart failure patients with sinus rhythm [8–12]. In an effort to reduce the incidence of ischaemic cerebrovascular accidents in LVAD patients, we have performed concomitant left atrial appendage closure (LAAC) at the time of LVAD surgery regardless of history of atrial fibrillation. The aim of this study is to evaluate the impact of concomitant LAAC on the incidence of ischaemic cerebrovascular accidents in LVAD patients.

## PATIENTS AND METHODS

### Patients and study design

Between January 2012 and November 2021, consecutive 310 patients underwent LVAD surgery with HeartMate II or 3 (Abbott Labs, Chicago, IL, USA) at our institution. Out of 310 patients, 98 patients (31.6%) underwent concomitant LAAC. The cohort was divided into 2 groups: patients with LAAC (group A, *n* = 98) and without LAAC (group B, *n* = 212). We compared the perioperative data and late outcomes between 2 groups. In the preoperative data, the cerebrovascular accident (CVA) was defined as whether a patient had a history of stroke or cerebral haemorrhage. In terms of antithrombotic therapy, all patients were initially treated with antiplatelet therapy and warfarin (a targeted international normalized ratio was 2.0–3.0) after LVAD implantation. The ischaemic cerebrovascular accident after LVAD implantation was defined as ischaemic stroke, haemorrhagic stroke or transient ischaemic attack with compatible clinical symptoms and/or diagnostic imaging. Diagnostic imagings were reviewed by experienced radiologists. Based on their review, cerebral haemorrhage which was not related with embolic stroke was excluded. A competing risk analysis based on the Fine and Gray method was used for estimation of the incidence of the ischaemic cerebrovascular accidents while taking competing risk event (death) into account. We also investigated if LAAC was associated with ischaemic cerebrovascular accidents by Fine and Gray proportional hazard regression analysis for competing risk event (death). This is a retrospective study. The institutional review board of our institution approved this study. All data including follow-up information were reviewed from each patient’s electric medical records. The mean follow-up period was 23.2 (22.1) months after LVAD implantation surgery. The mean follow-up index was 0.92 (0.2).

### Ethics statement

This study was approved by the institutional review board of our institution (IRB17-0413) on 21 December 2021 and written informed consent was waived by the institutional review board of our institution. This study complies with the ethical principles of the Declaration of Helsinki.

### Left atrial appendage closure

**Figure 1:**
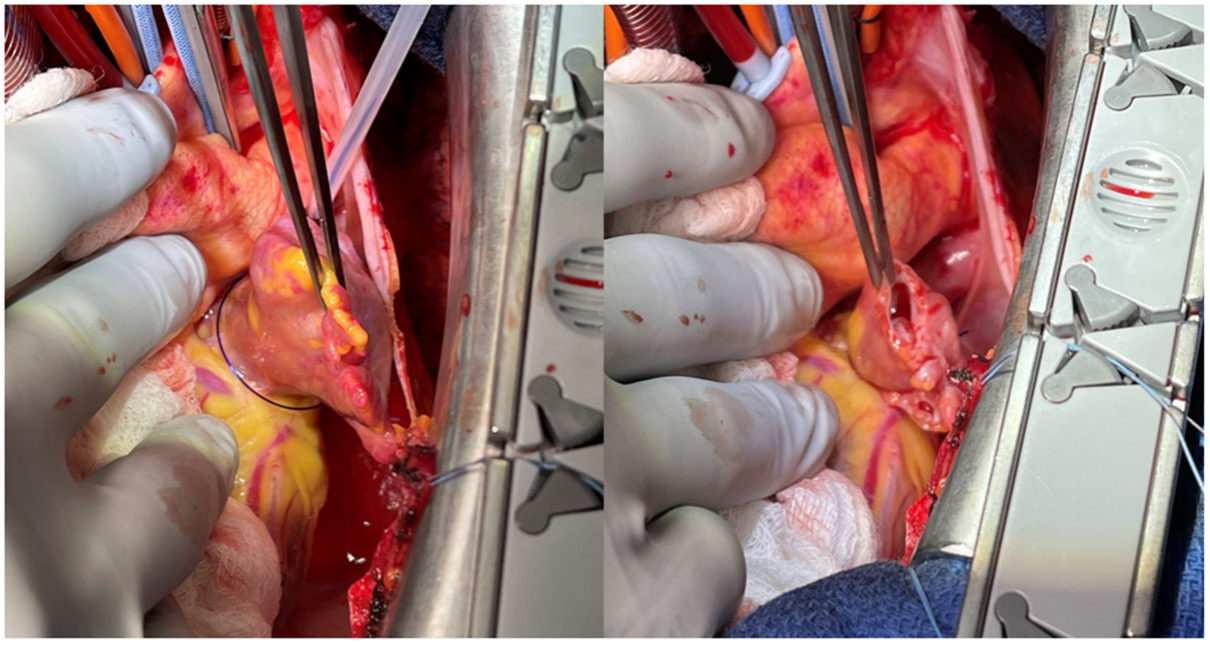
Intraoperative image of left atrium appendage closure using Endoloop ligature.

The LAA was assessed by the intraoperative transoesophageal echocardiography. If there is a thrombus in the LAA, we closed the appendage after removing the thrombus. LAAC was performed with the use of any of the following techniques: ligation using Endoloop ligature (Ethicon, Somerville, NJ), stapler closure using Endo GIA (Medtronic, Minneapolis, MN), or double-layer linear closure from within the left atrium or from outside the left atrium using 4–0 prolene suture. The indication of LAAC was based on multidisciplinary team decision and/or surgeon’s preference. A history of previous cardiac surgery was one of barriers for the LAAC because of the difficulty to access. Intraoperative transoesophageal echocardiography was used to confirm the thorough closure of the LAA. Intraoperative images of LAAC using Endoloop ligature are shown in Fig. 1.

### Statistical analyses

Statistical analysis was performed using JMP 11.0 software (SAS Institute Inc, Cary, NC, USA) and EZR version 1.61 software (Saitama Medical Center, Jichi Medical University, Saitama, Japan). Data were expressed as means (standard deviations) or median with interquartile range for continuous variables and as numbers (percentages) for categorical variables. Comparisons of continuous variables were tested with unpaired Student’s *t*-test or Wilcoxon test, and comparisons of categorical variables were tested with chi-squared test or Fisher’s exact test. Long-term survival was analysed using the Kaplan–Meier analysis and log-rank test. A competing risk analysis based on the Fine and Gray method was used for estimation of the incidence of the ischaemic cerebrovascular accidents while taking competing risk event (death) into account. The association between LAAC and ischaemic cerebrovascular accidents was analysed with Fine and Gray proportional hazard regression method for competing risk event (death). We included covariates which were rationally considered to be related with ischaemic cerebrovascular accidents such as CHADS2 score, LAAC, history of atrial fibrillation and HeartMate 3 implantation [1–6].

## RESULTS

### Preoperative data

Preoperative characteristics are detailed in Table 1. There were no significant differences in age, gender and body surface area. Baseline CHADS2 score was not significantly different between 2 groups (*P* = 0.19). History of atrial fibrillation was also not significantly different between group A (42.9%) and group B (42.5%) (*P* = 0.95). HeartMate 3 device was implanted more frequently in group A (53.1%) than in group B (40.1%) (*P* = 0.03). Group B had history of previous cardiac surgery more frequently than group A. There were no significant differences between the 2 groups in preoperative haemodynamic parameters.

**Table 1:** Preoperative characteristics

	All (*n* = 310)	GroupA (*n* = 98)	GroupB (*n* = 212)	*P*-Value
Age (years), mean (SD)	56.3 (13.6)	55.0 (12.3)	56.9 (14.1)	0.26
Female, *n* (%)	74 (23.9)	23 (23.5)	51 (24.1)	0.91
Height (cm), mean (SD)	174.5 (9.5)	174.4 (9.0)	174.6 (9.7)	0.87
Weight (kg), mean (SD)	94.7 (25.4)	95.6 (26.8)	94.3 (24.8)	0.66
BSA, mean (SD)	2.08 (0.3)	2.08 (0.3)	2.08 (0.3)	0.86
Hypertension, *n* (%)	223 (71.9)	67 (68.4)	156 (73.6)	0.34
Dyslipidaemia, *n* (%)	167 (53.9)	47 (48.0)	120 (56.6)	0.16
Diabetes mellitus, *n* (%)	131 (42.3)	38 (38.8)	93 (43.9)	0.40
CVA, *n* (%)	48 (15.5)	14 (14.3)	34 (16.0)	0.69
COPD, *n* ()	72 (23.2)	18 (18.4)	54 (25.5)	0.16
PVD, *n* (%)	27 (8.7)	3 (3.1)	24 (11.3)	<0.01
CHADS2 score, mean (SD)	2.52 (1.1)	2.40 (1.1)	2.58 (1.1)	0.19
IABP, *n* (%)	94 (30.3)	29 (29.6)	65 (30.7)	0.85
ECMO, *n* (%)	18 (5.8)	8 (8.2)	10 (4.7)	0.24
HeartMate 3, *n* (%)	137 (44.2)	52 (53.1)	85 (40.1)	0.03
History of atrial fibrillation, *n* (%)	132 (42.6)	42 (42.9)	90 (42.5)	0.95
Previous cardiac surgery, *n* (%)	75 (24.2)	5 (5.1)	70 (33.0)	<0.01
Antiplatelet, *n* (%)	161 (51.9)	49 (50.0)	112 (52.8)	0.64
Anticoagulation, *n* (%)	152 (49.0)	43 (43.9)	109 (51.4)	0.22
Creatinin (mg/dl), mean (SD)	1.56 (0.7)	1.56 (0.8)	1.56 (0.7)	0.98
LVDd (mm), mean (SD)	70.6 (11.5)	71.1 (11.4)	70.4 (11.6)	0.63
Ejection fraction (%), mean (SD)	19.7 (6.4)	19.6 (6.4)	19.7 (6.5)	0.87
Bridged to transplant, *n* (%)	49 (15.8)	16 (16.3)	33 (15.6)	0.46
CVP (mmHg), mean (SD)	13.8 (7.0)	14.5 (7.0)	13.5 (7.0)	0.26
Mean PAP (mmHg), mean (SD)	38.9 (11.1)	40.6 (11.1)	38.1 (11.0)	0.09
PAWP (mmHg), mean (SD)	26.4 (9.1)	27.5 (8.6)	25.9 (9.3)	0.18
PVR, mean (SD)	3.66 (2.5)	3.76 (2.5)	3.62 (2.5)	0.70
CI (l/min/m^2^), mean (SD)	1.89 (0.6)	1.91 (0.5)	1.89 (0.6)	0.70

BSA: body surface area; CI: cardiac index; COPD: chronic obstructive pulmonary disease; CVA: cerebrovascular accident; CVP: central venous pressure; ECMO: extracorporeal membranous oxygenation; IABP: intra-aortic balloon pump; LVDd: left ventricular end-diastolic diameter; PAP: pulmonary artery pressure; PAWP: pulmonary artery wedge pressure; PVD: peripheral vascular disease; PVR: pulmonary vascular resistance.

### Intraoperative data

Intraoperative data are shown in Table 2. Concomitant mitral valve repair was performed more in group A (51.0%) than in group B (32.1%) (*P* < 0.01). The cardiopulmonary bypass time and cross-clamp time were not significantly different between 2 groups.

**Table 2:** Operative data and early clinical outcomes

Variables	All (*n* = 310)	Group A (*n* = 98)	Group B (*n* = 212)	*P*-Value
Operative procedures, *n* (%)				
Aortic valve surgery	34 (11.0)	15 (15.3)	19 (9.0)	0.10
Mitral valve repair	118 (38.1)	50 (51.0)	68 (32.1)	<0.01
Tricuspid valve repair	133 (42.9)	38 (38.8)	95 (44.8)	0.32
CABG	20 (6.5)	2 (2.0)	18 (8.5)	0.02
Cardiopulmonary bypass time (min), mean (SD)	158.0 (55.9)	150.1 (48.2)	161.8 (59.0)	0.09
Cross-clamp time (min), mean (SD)	67.3 (31.5)	67.0 (23.3)	67.5 (35.4)	0.93
Intraoperative thrombus location, *n* (%)				
RA or PA	18 (5.8)	7 (7.1)	11 (5.2)	0.50
LA or LAA	7 (2.3)	6 (6.1)	1 (0.5)	<0.01
Left ventricle	45 (14.5)	16 (16.3)	29 (13.7)	0.54
Early clinical outcomes, *n* (%)				
In-hospital mortality	33 (10.7)	7 (7.1)	26 (12.3)	0.16
Haemodialysis	44 (14.2)	15 (15.3)	29 (13.7)	0.70
Tracheostomy	39 (12.6)	11 (11.2)	28 (13.2)	0.62
RVAD	37 (11.9)	15 (15.3)	22 (10.4)	0.22
Re-exploration for bleeding	35 (11.3)	9 (9.2)	26 (12.3)	0.18
ECMO	8 (2.6)	3 (3.1)	5 (2.4)	0.72
PT-INR at discharge, mean (SD)	2.17 (0.5) (*n* = 277)	2.20 (0.5) (*n* = 91)	2.16 (0.5) (*n* = 186)	0.56

CABG: coronary artery bypass grafting; ECMO: extracorporeal membranous oxygenation; LA: left atrium; LAA: left atrium appendage; PA: pulmonary artery; PT-INR: prothrombin time-international normalized ratio; RA; right atrium; RVAD: right ventricle assist device.

In group A, LAA was closed with Endoloop ligature in 88 patients (89.8%), double-layer linear closure from inside the left atrium in 8 patients (8.2%), from outside the left atrium in 1 patient (1.0%) and stapler closure in 1 patient (1.0%). Intraoperative transoesophageal echocardiography showed no residual communication between the left atrium and appendage in all cases.

### Early clinical outcomes

Early clinical outcomes are summarized in Table 2. Overall, in-hospital mortality was 10.7% (33/310). Postoperative complications included acute renal insufficiency newly requiring haemodialysis in 44 patients (14.2%), respiratory failure requiring tracheostomy in 39 patients (12.6%), right heart failure requiring right ventricle assist device in 37 patients (11.9%), re-exploration for bleeding in 35 patients (11.3%) and extracorporeal membranous oxygenation requirement in 8 patients (2.6%). There were no significant differences in hospital mortality and postoperative complications between 2 groups. Of note, in group A, 1 patient required re-exploration due to bleeding from LAA ligated with Endoloop ligature.

### Late outcome

During the follow-up period, 41 patients (13.2%) underwent heart transplantation and 2 patients (0.6%) underwent decommission. The survival rate in group A (87.5% at 12 months and 74.4% at 36 months) was not significantly different in group B (82.8% at 12 months and 71.9% at 36 months) (log rank = 0.16). At the latest follow-up, the number of patients on warfarin in group A (87.9%) was not significantly different from that in group B (83.3%, *P* = 0.31). In addition, international normalized ratio level in group A [2.28 (1.2)] was also not significantly different from that in group B [2.12 (1.2)] (*P* = 0.28).

### Cumulative incidence of ischaemic cerebrovascular accidents

**Figure 2:**
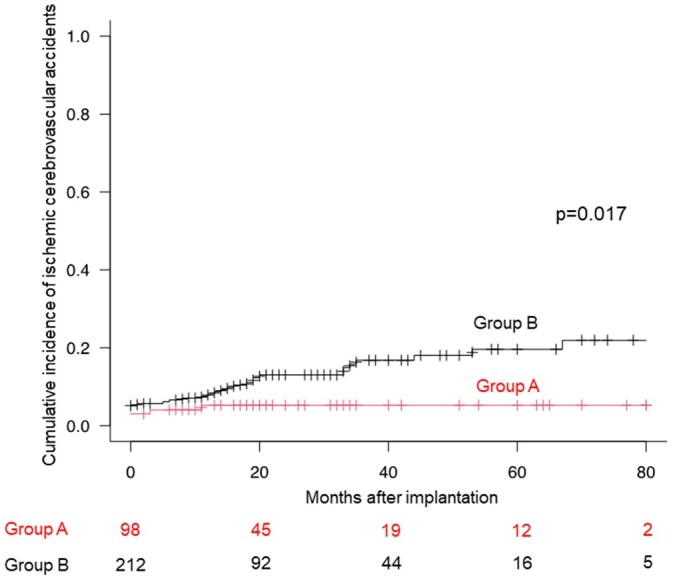
The cumulative incidence of ischaemic cerebrovascular accidents of the left atrial appendage closure group (group A) and non-left atrial appendage closure group (group B). The incidence of ischaemic cerebrovascular accidents in group A was significantly lower than that in group B.

Two patients in group A and 6 patients in group B developed perioperative ischaemic cerebrovascular accidents. Overall, 37 patients (11.9%) developed ischaemic cerebrovascular accidents during follow-up period. Ischaemic stroke occurred in 5 patients (5/98, 5.1%) in group A. In group B, there were ischaemic stroke in 24 patients (24/212, 11.3%), transient ischaemic attack in 7 patients (7/212, 3.3%) and heamorrhagic stroke in 1 patient (1/212, 0.5%). Six patients in group B were not on oral anticoagulation, and all patients in group A were on oral anticoagulation at the time of the occurrence of ischaemic cerebrovascular accidents. The cumulative incidence of ischaemic cerebrovascular accidents in group A (5.3% at 12 months and 5.3% at 36 months) was significantly lower than that in group B (8.2% at 12 months and 16.8% at 36 months) (*P* = 0.017) (Fig. 2). In the Fine and Gray proportional hazard regression analysis, LAAC was associated with reducing ischaemic cerebrovascular accidents (hazard ratio 0.38, 95% confidence interval 0.15–0.97, *P* = 0.043) (Table 3).

**Table 3:** The impact of left atrial appendage closure on ischaemic cerebrovascular accidents

Risk factors	Hazard ratio	95% confidence interval	*P*-Value
Left atrial appendage closure	0.38	0.15–0.97	0.043
CHADS2 score	1.38	1.06–1.80	0.018
History of atrial fibrillation	0.96	0.50–1.83	0.90
HeartMate 3	0.64	0.31–1.31	0.22

## DISCUSSION

This study demonstrated 2 major findings. First, the concomitant LAAC at the time of LVAD surgery was safely and efficiently performed without increasing perioperative mortality and complications. Second, patients who underwent concomitant LAAC experienced significantly less incidence of ischaemic cerebrovascular accidents than those who did not undergo LAAC. Multivariable analysis revealed that concomitant LAAC was an independent predictor to reduce the incidence of ischaemic cerebrovascular accidents.

### Ischaemic cerebrovascular accident in patients with left ventricular assist device

Ischaemic cerebrovascular accident is one of the significant complications in patients with LVAD. There are many published articles available about the incidence and risk factors for ischaemic cerebrovascular accidents in LVAD patients. Acharya *et al.* [1] analysed 7112 patients with continuous-flow LVAD from the INTERMACS database and reported that 10.6% experienced ischaemic cerebrovascular accidents during 9.79 months follow-up. A recent report from the MOMENTUM 3 trial including 361 patients with HeartMate II or 3 demonstrated that 14.4% experienced ischaemic cerebrovascular accidents [2]. Bravo *et al.* [3] also investigated 525 patients with HeartMate II or HeartMate 3 and showed that 8.2% experienced ischaemic cerebrovascular accidents during 6 months after LVAD surgery. Compared with these studies, the incidence of ischaemic cerebrovascular accident (11.9%) during follow-up period (23.2 (22.1)) months after LVAD in the present study was comparable.

A number of studies demonstrated that risk factors for cerebrovascular accidents included age, gender, a history of atrial fibrillation, implanted LVAD type, pump thrombosis and intracardiac thrombus [2–7]. However, it has not been well documented about the impact of concomitant LAAC at the time of LVAD surgery. The present study showed that the concomitant LAAC reduced the incidence of ischaemic cerebrovascular accidents significantly. Higher CHADS2 score might also be a significant predictor for the incidence of cerebrovascular accidents. In our multivariate analysis, HeartMate 3 implantation had a certain degree of impact on prevention of the ischaemic cerebrovascular accidents, while it did not reach to statistically significant difference. This might be due to relatively small sample size and limited follow-up period. In addition, it has to take into consideration that the patients with HeartMate 3 underwent more LAAC than those with HeartMate II. Although the existence of intracardiac thrombus and patients off warfarin at the latest follow-up were also analysed in another multivariate model, both of them were not a risk factor for the ischaemic cerebrovascular accidents in our cohort.

### The left atrial appendage is one of sources of thrombus in heart failure patients

The LAA is well known as a source of thrombus which causes a stroke in patients with atrial fibrillation. There are many studies about the impact of LAAC for patients with atrial fibrillation. A recent multicentre, randomized trial demonstrated that LAAC during cardiac surgery in patients with history of atrial fibrillation was able to reduce ischaemic stroke or systemic emboli events significantly [13]. Several investigators reported that the LAA was a source of thrombus not only in patients with atrial fibrillation, but also in heart failure patients without atrial fibrillation [8–12]. Kurzawski and colleagues investigated 63 patients with reduced ejection fraction (<25%) and without history of atrial fibrillation and showed that 20 patients (31.7%) had thrombi in LAA confirmed by transoesophageal echocardiography [12]. It is well accepted that there are some patients who develop new-onset atrial fibrillation after LVAD surgery. Hickey et al. reported that new-onset atrial fibrillation after LVAD surgery was 13% [14]. Hawkins *et al. also* reported that it was 17.6% in their study [15]. In our study, out of 178 patients without a history of atrial fibrillation, 45 patients (25.3%) experienced new-onset atrial fibrillation after surgery. Although little is known about the negative impact of new onset of atrial fibrillation after LVAD surgery, it could be a risk factor for ischaemic cerebrovascular accidents. Considering these reports, we believe that performing LAAC during LVAD surgery regardless of a history of atrial fibrillation would be reasonable.

### The left atrial appendage closure technique

There are several techniques to close the LAA: ligation, stapler closure, double-layer linear closure from inside/outside from the left atrium. In our study, the LAA was mostly ligated with the Endoloop ligature technique. This technique is quick, simple and cost effective. Knowing prolonged cardiopulmonary bypass time adversely affects clinical outcomes, we believe that the quick and simple technique would be suitable over the other techniques as double-layer linear closure requiring left atrial incision [16, 17]. Kimura *et al.* [18] reported the effectiveness of Endoloop ligature for LAAC using canine model showing that the internal surface of the ligated LAA was very smooth 1 month after LAAC and there was no residual LAA tissue in the left atrium.

Unlike ligation or linear closure, the stapler closure may have an adverse impact for LVAD patients as a bridge to heart transplantation. We experienced a heart transplant patient whose LAA was closed with a commercially available stapler from previous surgery. Due to significant adhesion around the stapler device, it was very challenging to take the recipient heart out without injuring pulmonary vein cuff. From our experience, the Endoloop ligature technique does not cause significant adhesion around the LAA area at the time of redo surgery [19]. While percutaneous closure devise could be another approach to close the LAA, we do not believe this would be a good option after LVAD placement since a percutaneous procedure requires the brockenbrough method that potentially causes a right-to-left shunt in LVAD patients [20]. It would be the best timing to perform concomitant LAAC at the time of LVAD surgery.

### Limitations

There are some limitations that need to be addressed. First, this is a retrospective and single-centre study. A prospective randomized study would be warranted to validate the findings in this study. Second, due to small sample size and short follow-up period in our cohort, the statistic power might be limited. Third, since the indication of LAAC was depended on multidisciplinary team decision and/or surgeon’s preference, and there was a tendency to skip a LAAC in patients with a history of cardiac surgery due to adhesion, there might have been a selection bias. Fourth, we do not have available data regarding if a patient had carotid artery stenosis or not before surgery, and new-onset atrial fibrillation during follow-up period, which might have impact on the outcome.

## CONCLUSION

Concomitant LAAC at the time of LVAD surgery can reduce ischaemic cerebrovascular accidents without increasing perioperative mortality and complications.

**Conflict of interest:** Sean Pinney is a consultant of Abbott and Medtronic. Valluvan Jeevanandam is a consultant of Abbott. Christopher Salerno is a consultant of Abbott, The other authors report no conflict of interest.

## Data Availability

Data are available on request.

## ABBREVIATIONS

LAAC: Left atrial appendage closure
LVAD: Left ventricular assist device
